# Viral Science: Masks, Speed Bumps, and Guard Rails

**DOI:** 10.1016/j.patter.2020.100101

**Published:** 2020-09-11

**Authors:** Ganesh Mani, Tom Hope

**Affiliations:** 1 Carnegie Mellon University, Pittsburgh, PA, USA; 2 Allen Institute for AI and the University of Washington, Seattle, WA, USA

## Abstract

The speed of science, especially while solving the recent pandemic puzzles, is causing concerns. We describe some salient issues as well as a framework for making the process of publishing, organizing, and retrieving scientific literature more efficient. Emerging building blocks leveraging AI, including natural language processing tools such as SciSight from the Allen Institute for AI, that permit faceted navigation and research group detection are highlighted.

## Main Text

With the world fixated on COVID-19, the WHO has warned that the pandemic response has also been accompanied by an *infodemic*: overabundance of information, ranging from demonstrably false to accurate. Alas, the infodemic phenomenon has extended to articles in scientific journals, including prestigious medical outlets such as *The Lancet* and *NEJM*. The rapid reviews and publication speed for COVID-19 papers has surprised many, including practicing physicians, for whom the guidance is intended. Politicians are adding to the maelstrom of confusion by touting speculative and unapproved treatments. The hydroxychloroquine saga has brought to focus the eager political push, which catalyzed rushed decisions by researchers, data aggregators, and the publishing venue.^1^ We are also likely to see continuing conflicts of opinions among scientists and politicians, with the rapid release of results from the numerous, ongoing vaccine trials. It is expected that multiple COVID-19 vaccine candidates will show promise, but they may differ along key dimensions—safety, efficacy, cost, country of origin, production, and delivery considerations—resulting in vigorous debates and potentially confusing citizens across the globe.

From a public policy standpoint, especially during the current COVID-19 crisis and in general, in normal times, there is a need to disseminate reliable information quickly. London and Kimmelman^2^ have recently argued against pandemic research exceptionalism, calling for the clinician and research community to resist the temptation of wide off-label use of untested interventions and the urge to forego controlled trials.

Such caveats are particularly important given the exponential growth in the number of preprints and shorter review cycles for peer-reviewed publications relating to disease transmission, treatment protocols, clinical trials, and economic impact. There were over 4,000 preprints in the (med,bio,Chem)arXivs by early May—which had, by mid-August, grown to over 8,000—containing the words “COVID-19” or “SARS-CoV-2,” while the average time to peer review and publish a journal article had shrunk from 117 to 60 days, across 14 titles in virology.^3^ Research relating to second-order pandemic effects, such as increase in depression due to quarantine or climate change data from decreased emissions, are also adding to the manuscript volume. On the other hand, historically, only roughly 14% of clinical trials result in approved drugs according to an MIT study.

Given this backdrop, we need to think about providing guard rails to viral science to advance the goals of robustness and reproducibility while disseminating research. Timely, reliable research is used to guide public policy decisions including investment outlays with significant economic impact and also to catalyze follow-on research and product development.

### A Candidate Framework

One of this article’s authors had proposed an electronic MEGAJOURNAL archive^4^ with “a hierarchical, pyramid structure; the apex being formed by the papers which go into today’s high-quality printed journals and the lowest … being formed by contributions that … constitute some of today’s conference and workshop papers.” It also envisaged interdisciplinary content pyramids via appropriate pointers to the papers in the areas constituting the interdisciplinary field. This was an early vision predating the worldwide web! Silos of knowledge are being broken down gradually, but the information overload continues.^5^ Scientists and potential collaborators are depending on social media and specialized, colleague-curated blogs to keep abreast of new research.

A contemporary, robust framework needs to consider the workflow plus associated data involved in the two major spheres of research: design and completion (largely internal to the group), as well as its dissemination, review, and archival (external, for the public). We suggest thinking about the data, workflow, and processes via three distinct phases: pre-publication, where the actors involved are the intra-group researchers; publication, where researchers have to interact with other actors such as reviewers and the editors; and post-publication, where actors include citizens, regulators, peer scientists, and consumers of the research.

Apart from distinct actors, the data and workflow in these phases will also vary. For instance, internal lab notebooks may also contain data relating to different interim steps and failed or stepping-stone experiments. Best practices for recording such items may help rapidly share the interim results and enhance reproducibility and verifiability, if questions come up during a review of the final data and results.

### Pre-publication

Science involves observation of things and processes, hypothesis formation (followed by falsification, confirmation, or revision), and documenting robust rules and insights. It is commonly accepted that scientists can spend up to a quarter of their working time searching for and reading relevant scientific literature to understand research methods and results therein, critically evaluating presented data. Finding research papers relevant to the investigational area and extracting pertinent information are frequently cited as challenges, particularly for scientists in training or a scientist crossing over from another discipline to do interdisciplinary work. Having pre-defined but dynamic ontologies (as the field evolves) and uniform metadata is one way to make the information retrieval efficient. Researchers should also use standardized terminology in their lab notes and interim reports, making it easier to use the well-accepted terms when they are ready to submit a manuscript externally to a conference or journal.

#### Defining Uniform Metadata for Manuscript Submission

While certain entities (e.g., gene sequences, chemical structures, plant taxonomies) have standardized metadata, much work remains to set standards around a significant number of scientific concepts, especially given the pace of discovery of new materials, processes, and insights.

For example, key metadata around a clinical trial may include the institution, scientist-physician names, kind of study, geography, cohort, ethical guideline, and IRB approval details along with method descriptions, gadgets, instruments, chemicals, and compounds used. It could also make clear (1) any statistical adjustments for known and measured confounders such as disease severity and site effects and (2) any potential conflicts of interest.

Likewise, field-specific ontologies should guide metadata regarding, for instance, new nanomaterials or novel machine-learning algorithms.

We also encourage progressing to a standardized two-track manuscript submission framework: (1) a human-readable version of the paper, with pre-ordained structure, and (2) a machine-readable version of the paper, using standardized terminology, ontology, and structure as much as possible, especially for the tables, figures, methods, and results exposition. This will enhance readability of the text version of the paper and complement any documented code that is deposited (e.g., in GitHub via Jupyter notebooks). Efforts like Papers With Code (part of Facebook AI) that have linked papers in the field of machine learning to source code and provided a leaderboard showing performance of algorithms on standardized datasets are early steps in this direction.

#### Identifying the Best Reviewers, Scheduling, and Providing *Prior Art* Searches

Editors working in tandem with specialized literature search AI engines (that permit faceted search guided by ontology to triage the submissions) can identify relevant reviewers and related or prior art.

The editor expects the reviewers to opine on novelty, replicability, lucidity, and whether the conclusions comport with presented data. The reviewer should check whether visualization (e.g., tables and figures, which again need to be standardized) supports manuscript clarity, comprehensibility, and comparability (with other related research). With recent developments in natural language processing (NLP), it might be possible to provide article metrics (e.g., number of articles related to the conclusion) and a scoring of grammar and style. The reviewers can also be pointed to any feedback or crowdsourced comments from the preprint archives.

### Publication

The goal of this phase should be 3-fold:

(1) Organizing the novel, technically sound, peer-reviewed addition to the literature, shedding light on context (thus, links to relevant, related research articles are key), authority, and vintage—e.g., calendar date may be known, but not whether the method or practice described has been subsumed by something new (in clinical practice, this could happen quickly; especially for novel diseases, as we are seeing in the current context).
(2) Enabling comparisons among “similar” research (separated by time or geography—sometimes it may have been published in a different language or in an unexpected or unconventional forum).
(3) Highlighting negative results (important in clinical practice but also relevant in nonclinical research, to avoid scientists going down the same blind alley, wasting precious resources). This last goal is currently under-emphasized in scientific publishing.

Paramount is ease of access to current best practice, state of the art, or efficient frontier of knowledge and, if clinical practice (or manufacturing process), making the flowchart or protocol available with updates at the point of care (or production).

The summary goal of this critical phase is to amplify novel, useful information and perhaps provide hints regarding its reliability (e.g., whether it is an initial, small-cohort study). Identifying information and research that are expected to be muted or subsumed by other larger studies is also key.

### Post-publication

The main goal here is to categorize and place the research on a spectrum ranging from “Retracted” to “Best practice or result” (e.g., additional notations codified as metadata can provide the reason for retraction or the duration a best practice has been in vogue, or the number of additional studies that support and re-validate it.)

Follow-up papers, critiques (e.g., community comments on PubPeer), editorials, and FDA adverse event reports should be linked to each paper, especially one that is widely followed for treatment protocols. Other information from social media, specialized information sites (e.g., RetractionWatch.com) and legal rulings may also be valuable to be linked and highlighted, but it may need to be curated with additional commentary providing context.

The steps above can be considered post-publication peer review, and lessons from the product ratings and reviews milieu may help. For instance, e-commerce sites attempt to tag whether a review is from a verified buyer. In spite of this, the fraction of fake reviews is high, and thus, care must be taken to provide guard rails (e.g., by tracking the authority and affiliation of the reviewer).

### Automation and Human Curation

Given the ever-increasing research volume, it will be hard for humans alone to keep pace. Research generation has been amplified by computers embodying Moore’s law, and as that abates, expect advances in quantum computing to step in. A jujutsu-esque move is called for, using computers imbued with NLP and other AI techniques to organize and vet the research, ensuring appropriate governance standards (via human judgement, when apropos, as bias-free AI is still a work in progress).

### NLP and AI Assist: An Example

Scientists typically search and consume literature via lists of articles in academic search engines such as Google Scholar or Semantic Scholar. Search engines are good at quickly finding documents relevant to a specific targeted query. Many institutions released a flurry of literature search tools in the wake of COVID-19, a majority of which focused on academic search interfaces (https://covid19-research-explorer.appspot.com/, https://covid19search.azurewebsites.net/) and question answering (https://cord19.aws/, https://covidscholar.org/). More useful are tools that permit searching and filtering by various facets, e.g., specific journals or chemical mentions. The NIH’s LitCovid (https://www.ncbi.nlm.nih.gov/research/coronavirus/) includes as facets countries, journals, and chemicals and categorizes papers into areas such as whether they discuss treatments or mechanisms. Using NLP-driven automation to extract knowledge, including evidence-based interventions, from clinical trial publications is also starting to get some attention (https://bwallace.github.io/evidence_inference/). Other emerging tools based on AI include those from WellAI and Scite.ai. The offering from Scite.ai (https://scite.ai/visualizations/covid) helps decorate references with *affect*: denoting whether they provide contradicting or supporting evidence, or are simply citing (neutral).

But what about the things researchers are unaware of? When, in lieu of a specific query, they want to explore a general area or find out about new connections. Search engines are less useful for making connections that are not obvious from reading individual documents and thus are often difficult to use for exploration. Previous research work on coronaviruses and more broadly in biology and medicine may contain valuable knowledge for connecting the dots between past knowledge and new research. This wealth of information represents a great opportunity, but also a challenge: keeping up, managing the information overload,^5^ and making sure we have the most pertinent knowledge readily accessible. COVID-19 lends special urgency to this long-standing problem, as knowledge sharing across labs is required.

### SciSight

The Allen Institute for AI (AI2) and Semantic Scholar launched the COVID-19 Open Research Dataset (CORD-19), a growing corpus of papers (currently 130,000 abstracts plus full-text papers being used by multiple research groups) that are related to past and present coronaviruses.

**Figure 1:**
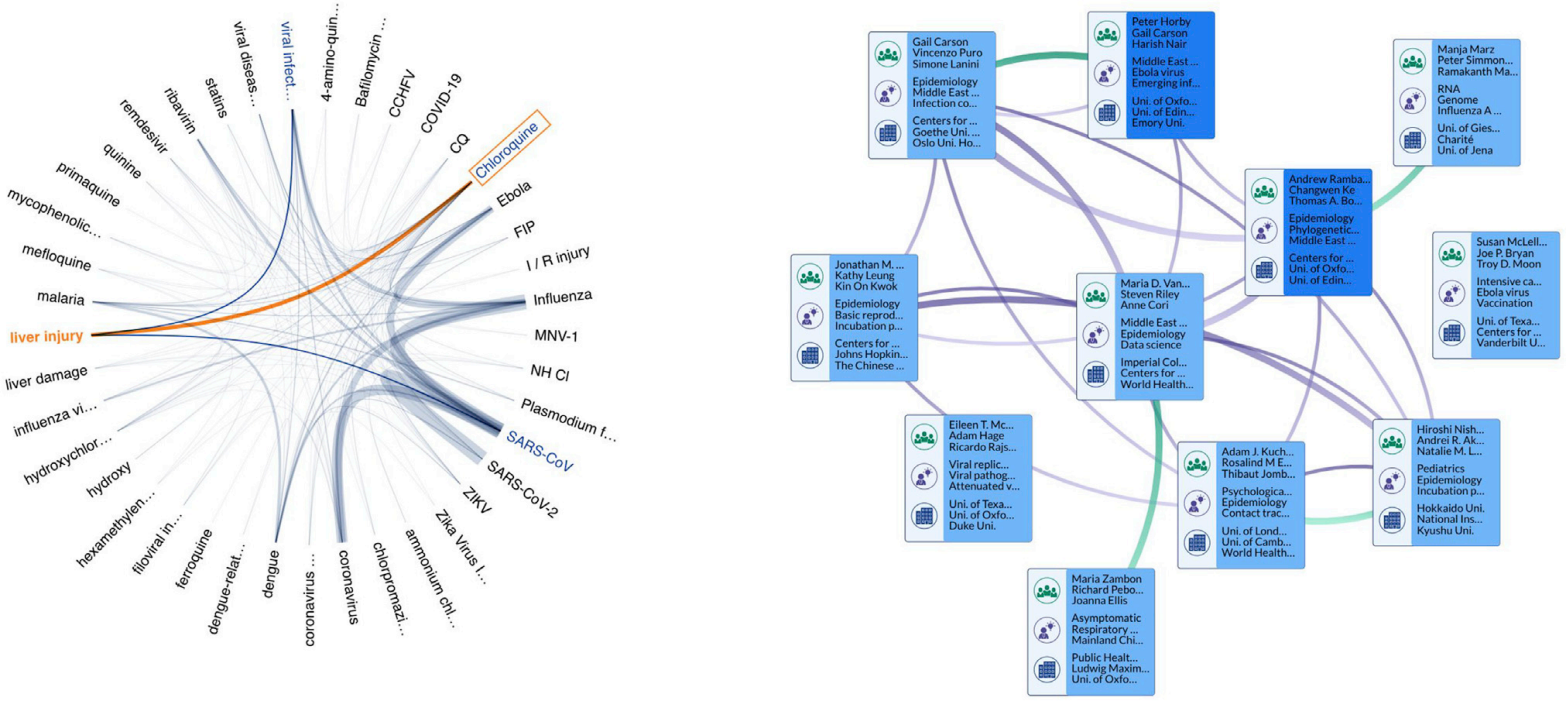
Associative Exploration (via Different Facets) in SciSight

Using this data, AI2, working with the University of Washington, released a tool called SciSight, an AI-powered graph visualization tool enabling quick and intuitive exploration^6^ of associations between biomedical entities such as proteins, genes, cells, drugs, diseases, and patient characteristics as well as between different research groups working in the field. It helps foster collaborations and discovery as well as reduce redundancy. For example, in Figure 1, left, the network of diseases and chemicals associated with “chloroquine” is displayed, as seen in SciSight.

Users can also find new groups and what directions they are working on by searching topics, affiliations, or authors, as shown in Figure 1, right.

### Call to Action

Information integrity is under attack. A preview of the inimical effects can be found on social media with respect to general news. Strong medicine might be required to prevent the spread of “Twitterenza” to scientific literature!

We believe—especially in light of the rapid increase in research production volume—that new standards need to be created around metadata (for indexing and retrieval) and review processes made more robust and transparent. Best practices for statistical validation may need to be revisited and communicated clearly to scientists and physicians (e.g., for clinical trials).

The research community and scientific publishers working together need to develop and make accessible open-source software tools to permit the dual-track submission discussed above. Repositories such as Github are a start, but there is a need for more features and decorations to make facile some of the goals we have discussed (e.g., code and data that are congruent with the latest protocol for a patient with a certain disease and comorbidity).

Others have called for^7^ and a few journals have started publishing peer reviews; however, even when reviews are published, many withhold reviewer identities. Much like cross-examination proceedings in a court of law, we believe publishing reviews along with reviewer identities can enhance transparency and build trust. Again, standard formats, including metadata, will help reliably automate the process of extracting and aggregating reviewer sentiment; this is another important building block in the proposed overall architecture.

There is a bias in science toward positive results; much of published research is prone to *overfitting*. Highlighting and publishing negative results can be a counterbalance; it will also have the salubrious effect of informing other scientists’ research agendas and prevent them from going down the same blind alleys.

Cooperation among publishing venues may be required to actualize the vision described herein (e.g., rating and sharing reviewers among journals, cross-referencing research, standardizing APIs for things such as dual-track submissions). Also, reviewers should be given career credit based on the quantity and quality of their reviews.

In conclusion, we are proposing an ambitious framework (partly summarized in Table 1) to start a dialog around it, dovetailing with the emerging field, “SciSci” (Science of Science^8^). Our framework involves standards for structuring, reviewing, and governing scientific knowledge plus a methodology to keep scientific information current and accurate—by weeding out stale knowledge and practices, by helping retract or fade to the background questionable work, honest mistakes, impoverished experiments, and fog-of-war protocols (that are sometimes necessary, like during the current COVID-19 crisis). Some of the focused scientific knowledge-engineering research efforts that have recently gained momentum—such as the aforementioned SciSight modules and CovidScholar—are interesting building blocks that give us a head start toward this grand goal of making science less tendentious. However, to actualize the vision above, we need a complete set of deliberately designed building blocks as well as a protocol for human experts and machines to collaborate in this milieu. Putting such infrastructure in place will help society with the next strategic surprise or grand challenge, a solution to which is likely to be equally, if not more, knowledge intensive.

**Table 1:** Summary Recommendations by Publication Phase

**Phase**	**Recommendation**
Pre-publication	Extract metadata based on standardized ontology (and dictionary)
	Encourage two-track manuscript submission: human-readable and machine-readable (not just code)
	Identify best reviewers and provide prior art searches (best done by editor)
Publication	Organize new paper in the context of other literature, enabling “compare and contrast” with related work
	Identify as novel or supporting existing work (boosting confidence in replicability)
	Highlight negative results
Post-publication	Facilitate post-publication peer comments
	Annotate paper with any critiques, link to newer (related) papers
